# Simultaneous Occurrence of Buckle Infection and Migration: A Case Report

**DOI:** 10.3390/medicina59030449

**Published:** 2023-02-23

**Authors:** Yasuyoshi Motose, Hiroto Terasaki, Misaki Ichiki, Mahono Okawa, Naohisa Mihara, Narimasa Yoshinaga, Taiji Sakamoto

**Affiliations:** Department of Ophthalmology, Graduate School of Medical and Dental Sciences, Kagoshima University, Kagoshima 890-0065, Japan

**Keywords:** buckle infection, buckle migration, scleral buckling

## Abstract

*Background*: When scleral buckling is performed using a #240 encircling band anterior to the equator for rhegmatogenous retinal detachment, buckle migration may occur anteriorly, eroding the rectus muscle. There are few cases of buckle migration occurring simultaneously with buckle infection. Notably, most previous reports included inadequate data on the pathophysiology of buckle migration and did not include the Hess test and perioperative images. *Case presentation*: A 36-year-old man with a history of atopic dermatitis underwent scleral buckling for rhegmatogenous retinal detachment of the left eye with #287 and #240 encircling bands at Kagoshima University Hospital. Four years later, he developed discharge, redness, and diplopia of the left eye. He was then referred to our hospital because buckle infection was suspected. The buckle was partially visible on the lower nasal side. Optical coherence tomography of the anterior chamber revealed the buckle to be on the nasal side and overlying the medial rectus muscle. Buckle migration and infection in the left eye was diagnosed, and early buckle removal was recommended. Two weeks later, on the day before surgery, conjunctival melting progressed in the nasal and inferior areas, and the buckle was exposed to a greater extent. In the surgical video at the initial surgery, the silicone band was confirmed to pass under the four rectus muscles, specifically the inferior and medial rectus muscles. At the beginning of the second surgery, we confirmed that the buckles were over the inferior and medial rectus muscles. As far as could be observed after buckle removal, the inferior and medial rectus muscles were not present at the normal location. Postoperatively, ocular pain and discharge quickly resolved. The subjective symptoms of diplopia also improved, and the postoperative Hess chart showed an improved ocular movement in the upward and lateral directions. *Conclusions*: Buckle migration is a rare postoperative complication of scleral buckling; however, patients at risk of buckle migration, such as those with encircling scleral buckle anterior to the eyeball, should be monitored with caution. If a buckle infection develops, buckle migration may occur within a short period, and early buckle removal should be considered.

## 1. Background

In some cases of scleral buckling performed for rhegmatogenous retinal detachment, proliferative vitreoretinopathy and proliferative diabetic retinopathy using a #240 encircling band anterior to the equator, the buckle may shift anteriorly after surgery, erode the rectus muscle, and migrate anteriorly to the muscle attachment site. This phenomenon is called buckle migration [1,2]. Although buckle migration has been often reported [3,4,5,6,7], its simultaneous occurrence with buckle infection has rarely been reported.

Here, a case of buckle migration and infection occurring 4 years after primary retinal detachment by scleral buckling with a #240 encircling band has been reported.

## 2. Case Presentation

A 36-year-old man with a history of atopic dermatitis underwent scleral buckling for rhegmatogenous retinal detachment of the left eye with #287 and #240 encircling bands at Kagoshima University Hospital 4 years ago. After the surgery, the retina remained attached, and he was followed up at our outpatient clinic for 2 years, following which his visits stopped.

**Figure 1:**
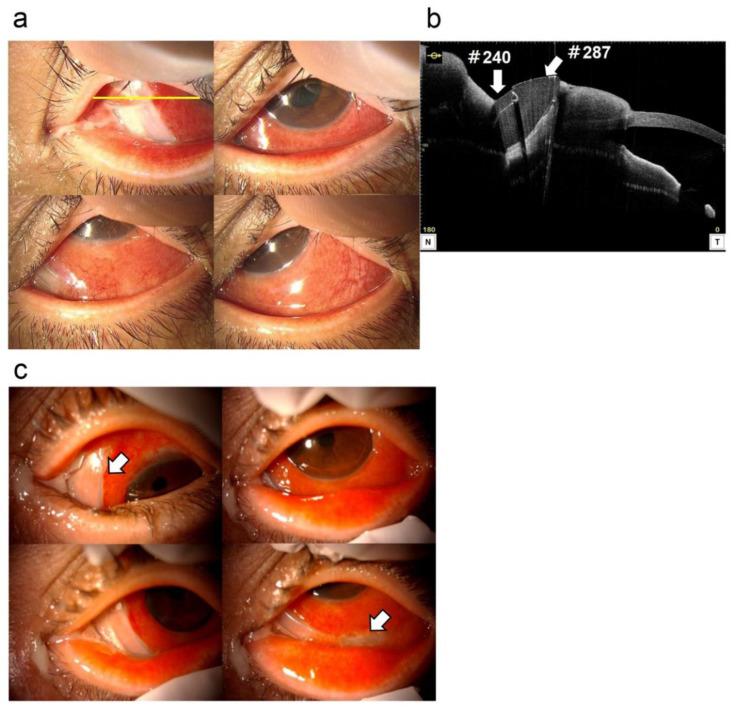
Preoperative anterior segment findings at the initial visit. Ophthalmoscopy revealed hyperemia, conjunctival edema, and discharge of the left eye at the primary visit. The cornea was clear, and there were no findings suggestive of inflammation in the anterior chamber or vitreous cavity. The buckle is partially revealed outside the eye on the lower nasal side (**a**). This was confirmed using anterior chamber optical coherence tomography (**b**) the yellow line in Figure 1a was scanned). The #240 silicone band and #287 buckle were revealed and lied over the medial rectus muscle on the nasal side. Two weeks later, on the day before surgery, conjunctival melting progressed in the nasal and inferior areas, and the buckle was exposed to a greater extent (**c**).

In October of X year, he developed discharge, redness, and diplopia of the left eye. He then visited his local ophthalmologist, who diagnosed him with scleritis of the left eye and referred him to Kagoshima University Hospital; however, the patient refused the visit. On November 22, he visited the same ophthalmologist again because his symptoms showed no improvement. He was then referred to the Department of Ophthalmology at Kagoshima University Hospital because buckle infection was suspected. At the time of the initial visit to the university hospital, the patient’s visual acuity was 1.0 (1.2 × S − 0.5 D) in the right eye and 0.8 (0.9 × C − 1.0 D A 180°) in the left eye, and his intraocular pressure was 13 mmHg in the right eye and 10 mmHg in the left eye. Ophthalmoscopy revealed hyperemia, conjunctival edema, and discharge of the left eye. The cornea was clear, and there were no findings suggestive of inflammation in the anterior chamber and vitreous cavity. The buckle was partially visible on the lower nasal side (Figure 1a). Optical coherence tomography (OCT) of the anterior chamber revealed the buckle to be on the nasal side and overlying the medial rectus muscle (Figure 1b).

**Figure 2:**
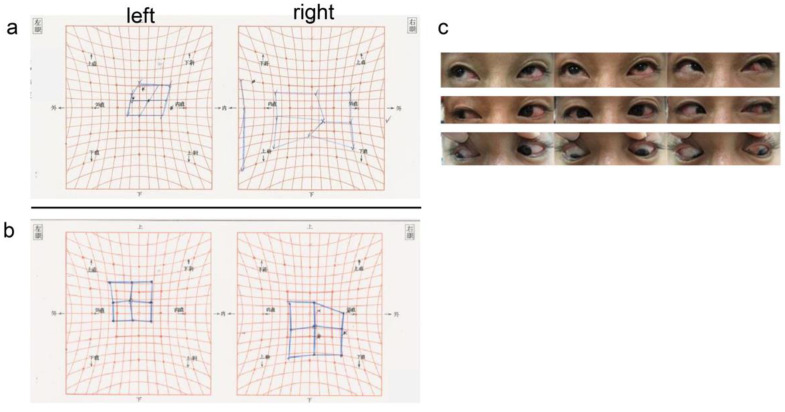
Preoperative and postoperative Hess charts. The Hess test showed limitation of eye movements in all directions, especially in abduction and inferior rotation of the left eye. (**a**) After buckle removal, subjective symptoms of diplopia improved, and the postoperative Hess chart showed improvement in ocular motility in the upward and lateral directions (**b**,**c**).

The Hess chart showed limitation of the eye movement in all directions, especially in abduction and inferior rotation of the left eye (Figure 2a). A fundus examination of the left eye showed no vitreous opacity, suggesting intraocular inflammation. The retina was reattached, but buckle elevation was not observed.

Buckle migration and infection in the left eye was diagnosed, and early buckle removal was recommended. However, at the patient’s request, the buckle removal procedure was scheduled for 2 weeks later. On the day before surgery, conjunctival melting progressed in the nasal and inferior areas, and the buckle was exposed to a greater extent (Figure 1c).

### 2.1. Intraoperative Findings at Initial Surgery and Buckle Removal

#### 2.1.1. Initial Surgery

Four years ago, scleral buckling was performed with #287 and #240 silicone encircling bands at Kagoshima University Hospital. During the surgery, the conjunctiva was dissected along the corneal rim, and the conjunctiva/tenon sac and sclera were separated to expose the rectus muscles.

**Figure 3:**
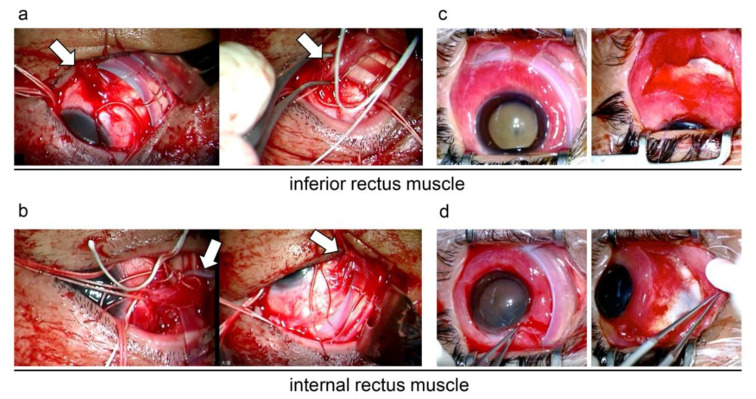
Intraoperative findings at the initial surgery and buckle removal. During the initial surgery, the silicone band was confirmed to pass under the four rectus muscles, specifically the inferior (**a**, white arrow) and medial rectus muscles (**b**, white arrow). Before buckle removal, the buckles were verified to be over the inferior (**c**, left) and medial rectus (**d**, left) muscles. After buckle removal, the inferior (**c**, right) and medial rectus (**d**, right) muscles were not present at the normal location.

Because the causative retinal tear was located at the ora serrata from the 7 to 11 o’clock position, #240 and #287 silicone bands were used. The silicone band was confirmed to pass under the four rectus muscles, specifically the inferior and medial rectus muscles (Figure 3a,b).

#### 2.1.2. Buckle Removal

Before the surgery, buckles were verified to be over the inferior and medial rectus muscles (Figure 3c,d).

The conjunctiva was first incised along the corneal limbus from above, and Tycron sutures at the 10 o’clock position were removed. The #287 and #240 buckles were pulled from the exposed areas and removed. As far as could be observed after buckle removal, the inferior and medial rectus muscles were not present at the normal location (Figure 3c,d). All Marcelline sutures holding the buckles in place were removed, and the conjunctiva was sutured after thorough sterilization of the subconjunctival area. The removed buckles were subjected to bacterial culture, and methicillin-susceptible *Staphylococcus aureus* (MSSA) was detected.

Postoperatively, ocular pain and discharge quickly resolved. Subjective symptoms of diplopia also improved, and the postoperative Hess chart showed improved ocular movement in the upward and lateral directions (Figure 2b,c).

## 3. Discussion

Risk factors for buckle migration include performing encircling on the anterior side rather than at the equator, high buckles, improper anchor sutures to the sclera, and degeneration of the sclera due to extensive cryocoagulation [1,2]. In the present case, the causative retinal tear was located at the ora serrata from the 7 to 11 o’clock position in the initial retinal detachment. Thus, encircling was performed on the anterior side rather than at the equator, thus causing buckle migration.

There have also been reports of buckle migration after head trauma [4]. Thus, chronic mechanical irritation due to atopic dermatitis may have been an inciting factor in the current case. Indeed, the most common causative agent of blepharitis in patients with atopic dermatitis is *Staphylococcus* species [8]. MSSA infection after encircling has been reported in 18.8% of patients with atopic dermatitis, but only in 0.4% of patients without atopic dermatitis [9]. Because MSSA was the causative bacterium in the present case, the present event was considered to be related to atopic dermatitis.

To date, only two cases of simultaneous occurrence of buckle migration and infection have been reported, and causative organisms were identified [5,7]. One was a postoperative case of retinal detachment associated with atopic dermatitis as in the present case [7]. Disease onset occurred 3 weeks after the initial surgery and was accompanied by necrotizing scleritis, suggesting that buckle migration occurred secondary to an acute postoperative infection. This suggests that inflammation due to buckle infection may cause buckle migration. In the present case, conjunctival melting and buckle migration progressed over a 2-week period from the initial visit until buckle removal, suggesting that buckle infection may promote buckle migration.

Regarding ocular motility disorders and diplopia, previous reports have shown that some patients experience diplopia before surgery, while others do not. In most reports, diplopia improved after buckle removal, and prognosis was favorable [1,2].

Possible reasons of favorable improvement in diplopia after surgery include the following: first, when the rectus muscle is slowly eroded by the buckle, muscle attachment is temporarily disrupted; however, after the buckle overcomes the rectus muscle, it reattaches to the sclera and rectus muscle retains its function. Second, the mechanical restriction of eye movement by the buckle, which is shifted from its original position, is released, causing an improvement in eye movement [1,2]. In the present case, the improvement in diplopia was most likely due to the release of the mechanical restriction of the eye movement by the migrating buckle, considering that eye movement improved in all directions when comparing the preoperative and postoperative Hess charts.

There have been many reports and discussions of buckle migration, but only few reports have shown examination results including the preoperative and postoperative Hess chart and surgical findings, as in the present case.

In the present case, it was objectively demonstrated that the removal of the migrating buckle improved diplopia, as discussed in the literature. In addition, preoperative anterior OCT and surgical findings revealed that the buckle, which surely passed under the rectus muscle in the initial surgery, migrated anteriorly to the inferior and medial rectus muscles and that the attachment sites of the inferior and medial rectus muscles were not present in their original positions.

## 4. Conclusions

In summary, buckle migration is a rare postoperative complication of scleral buckling; however, patients at risk of buckle migration, such as those with encircling scleral buckle anterior to the eyeball, should be monitored with caution. If a buckle infection develops, buckle migration may occur within a short period, and early buckle removal should be considered.

## Data Availability

Data sharing is not applicable to this article as no datasets were generated or analyzed during the current study.

## Abbreviations

OCT	Optical coherence tomography
MSSA	Methicillin-susceptible *Staphylococcus aureus*
